# Regional Differences in Cardiac Marker Gene Expression and Branched-Chain Amino Acid Metabolism in the Bovine Heart

**DOI:** 10.3390/ani16132014

**Published:** 2026-07-01

**Authors:** Rin Takiguchi, Kenichi Watanabe, Yutsuki Doai, Misuzu Hashimoto, Hiroyuki Watanabe, Yuki Muranishi

**Affiliations:** 1 School of Agriculture and Animal Science, Obihiro University of Agriculture and Veterinary Medicine, Obihiro 080-8555, Hokkaido, Japan; s25170019@st.obihiro.ac.jp (R.T.); knabe@obihiro.ac.jp (K.W.); hiwatanabe@obihiro.ac.jp (H.W.); 2 Laboratory for Molecular and Developmental Biology, Institute for Protein Research, The University of Osaka, Suita 565-0871, Osaka, Japan

**Keywords:** cardiac development, gene expression, principal component analysis

## Abstract

Although the hearts of large animals are known to exhibit regional heterogeneity in structure, function, and metabolism, their spatial patterns of gene expression remain poorly understood. Therefore, we investigated regional differences in gene expression across six anatomical regions of the bovine heart, including the atria, ventricles, interventricular septum, and apex (*n* = 3). The expression levels of genes related to cardiac development, structure, and branched-chain amino acid (BCAA) metabolism were analyzed using quantitative real-time PCR, and multivariate patterns were evaluated using principal component analysis (PCA). Histological analysis suggested no apparent structural differences among the examined regions. *NKX2.5* expression showed no significant regional variation, suggesting conserved cardiomyocyte identity. Conversely, contractile genes (*MYH7*, *MYL2*, *TNNI3*, and *TNNT2*) and the BCAA metabolism-related gene *BCKDHA* were significantly more highly expressed in the ventricular myocardium than in the atrial regions. PCA clearly distinguished between the atrial and ventricular myocardium and indicated coordinated variations in contractile and metabolic gene expression. These findings of region-specific molecular heterogeneity in the bovine heart, characterized by regional differences in gene expression across the myocardium rather than morphological differences, provide new insights into the spatial regulation of cardiac function and highlight the value of the bovine heart as a model for studying myocardial specialization.

## Simple Summary

Rather than being a uniform organ, the heart exhibits important function-related differences among its various regions. However, the molecular mechanisms that regulate these regional differences in large animals remain to be fully elucidated. In this study, we examined gene activity in different regions of the bovine heart, including the atria, ventricles, interventricular septum, and apex. We found that genes involved in muscle contraction and energy metabolism were more active in the ventricles than in the atria, whereas no clear structural differences were observed between the two regions. These results indicate that functional differences in the heart are mainly associated with molecular changes rather than visible structural dissimilarities. Our findings also suggest that gene activity differs among anatomical regions of the heart. This study contributes to a better understanding of the regional differences in heart function and contributes to a better understanding of cardiac biology in cattle.

## 1. Introduction

The heart is a highly complex organ composed of four morphologically and functionally distinct chambers that operate under markedly different hemodynamic conditions, including variations in blood pressure and mechanical load. These chamber-specific environments impose distinct mechanical, electrical, and metabolic demands on cardiomyocytes, leading to substantial regional specialization within the heart [1,2,3]. In humans, the atrial and ventricular myocardium differ in developmental origin, structural organization, hemodynamic load, and physiological properties, which are reflected in their distinct chamber-specific gene expression profiles [4].

In recent years, transcriptomic approaches, including bulk RNA sequencing and single-cell analyses, have been increasingly used to elucidate the regional differences in cellular composition, molecular characteristics, and functional properties within the heart [5]. Comparative transcriptomic studies of atrial and ventricular cardiomyocytes have provided compelling molecular evidence that these muscle cells do not constitute a homogeneous population [4,6]. However, knowledge regarding spatial differences in the expression of genes critical for cardiac function, including those examined in the present study, remains limited. Although such regional specialization is essential for efficient and coordinated cardiac performance, it also underlies region-specific vulnerability to various types of pathological stress, including ischemia, hypertrophy, and heart failure [4].

Consistent with this regional specialization, cardiomyocytes exhibit distinct gene expression patterns that correlate with their functional roles. In humans, the myosin heavy chain 7 (*MYH7*) and myosin light chain 2 (*MYL2*) genes are highly expressed in the ventricular myocardium, whereas the myosin light chain 4 (*MYL4*) gene is preferentially expressed in the atrial myocardium [4,6]. Similarly, in mice, the actin alpha cardiac muscle 1 (*Actc1*), cardiac troponin T2 (*Tnnt2*), and tropomyosin 1 (*Tpm1*) genes are highly expressed across all cardiac chambers, whereas *Myl4* is predominantly expressed in the atria and *Myl2* and *Myl3* are mainly enriched in the ventricles, indicating pronounced regional regulation of cardiac gene expression in the heart [7]. Among these regionally regulated genes, *Nkx2.5*, which encodes the cardiac-specific transcription factor NK2 homeobox 5, plays a central role in the complex regulatory program that governs cardiac development [8]. *Nkx2.5* is expressed from the embryonic stage through to adulthood, and gradients in its expression during development are known to contribute to the formation of the atria and ventricles [8,9]. However, despite the well-established roles of *Nkx2.5* in cardiac development, the regional differences in its expression within the heart remain poorly understood.

The regional heterogeneity of cardiomyocytes is also reflected in metabolic differences across the heart. Previous studies on rats and rabbits have reported dissimilarities in glycogen content and myosin ATPase activity between the atrial and ventricular myocardium, suggesting distinct metabolic and contractile properties across the cardiac regions [10,11]. Moreover, one study showed that myocardial fatty acid uptake and oxygen consumption were higher in regions with higher blood flow [12]. Although studies of myocardial energy metabolism have predominantly focused on fatty acid oxidation and glucose metabolism, which are the principal energy-producing pathways in the heart, the cardiac metabolism of branched-chain amino acids (BCAAs) has attracted increasing attention in recent years [13]. However, despite accumulating evidence of regional metabolic heterogeneity at the functional level, relatively few studies have systematically examined region-specific differences in the expression of genes involved in these metabolic pathways. In addition to chamber-specific differences, previous studies have suggested regional variations in mechanical load, oxygen demand, and metabolic activity across the heart.

Cardiac regional heterogeneity has been studied predominantly in humans and rodent models [14,15]. In contrast, systematic investigations of regional differences in myocardial gene expression and metabolism remain limited in large animal species. It remains unclear whether comparable regional differences exist in the bovine myocardium, particularly with respect to genes involved in cardiac contractile function and BCAA metabolism. The bovine heart offers a particularly advantageous model for investigating regional specialization, as its large size and well-defined anatomical architecture facilitate detailed comparisons among distinct cardiac regions [16]. We therefore hypothesized that molecular heterogeneity exists across anatomically distinct regions of the bovine myocardium and that genes involved in contractile function and BCAA metabolism exhibit region-specific expression patterns.

Therefore, in this study, we aimed to characterize the regional specialization of gene expression in the bovine heart. Specifically, we analyzed regulatory genes in cardiac development, major cardiomyocyte structural markers, and genes involved in BCAA metabolism across the atrial and ventricular regions, including the interventricular septum (IVS) and apex (Apex). Our results suggested region-specific differences in gene expression within the bovine myocardium. These findings provide valuable insights into region-specific myocardial function and molecular specialization in the bovine heart.

## 2. Materials and Methods

### 2.1. Animals and Cardiac Tissue Sampling

Hearts from the carcasses of Holstein and F1 crossbred cattle (2 weeks to 5 months of age, of both sexes) were provided by the Division of Veterinary Sciences, Section of Anatomy and Pathology, Obihiro University of Agriculture and Veterinary Medicine (Supplementary Table S1). None of the hearts showed gross pathological abnormalities on macroscopic examination.

To evaluate regional gene expression levels, cardiac tissue samples were collected from six anatomically distinct regions of the heart, namely, the right atrium (RA), left atrium (LA), right ventricle (RV), left ventricle (LV), interventricular septum (IVS), and apex (Apex) (Supplementary Figure S1). Tissue samples from all six regions were obtained from each of three animals and washed with phosphate-buffered saline to remove residual blood. For molecular analysis, approximately 100 mg of tissue was homogenized using a high-speed homogenizer (HisCotron, Microtec, Chiba, Japan) and stored at −80 °C until further processing. For histological evaluation, parallel tissue specimens (~1 cm^3^) were fixed in 10% neutral-buffered formalin as described below.

### 2.2. Histological Analysis

Cardiac tissue samples were first fixed in 10% neutral-buffered formalin and then dehydrated using a graded ethanol series, cleared in xylene, and embedded in paraffin wax. The paraffin-embedded tissues were sectioned at approximately 4 µm thickness using a microtome (REM-700, Yamato Kohki, Niiza, Japan). The sections were mounted on glass slides, air-dried, deparaffinized, and rehydrated. Subsequently, the sections were stained with hematoxylin and eosin (H&E) according to standard histological protocols. The tissue sections were examined using an inverted microscope (OPTIPHOT-2, Nikon, Tokyo, Japan), and images were captured with a digital camera (Digital Sight 1000, Nikon) at ×200 and ×400 magnifications.

### 2.3. RNA Preparation and Quantitative Real-Time PCR

Total RNA was extracted from the tissue samples using the TRIzol reagent (Thermo Fisher Scientific, Waltham, MA, USA), and the concentration was measured using a DeNovix spectrophotometer (DS-11, DeNovix, Wilmington, DE, USA). Then, 1 µg of the RNA was treated with DNase and reverse-transcribed into cDNA using random primers (48190011, Thermo Fisher Scientific) and SuperScript II (18064022, Thermo Fisher Scientific) on the GeneAtlas Thermal Cycler 482 system (4990902, ASTEC, Kasuya, Japan). Quantitative real-time PCR (qPCR) was performed using the SsoAdvanced Universal SYBR Green Supermix (1725271, Bio-Rad, Hercules, CA, USA) and a LightCycler 96 system (05815916001, Roche, Basel, Switzerland) according to the manufacturer’s instructions. The PCR conditions consisted of an initial denaturation step at 95 °C for 30 s, 40 cycles of denaturation at 95 °C for 10 s, and annealing/extension at 60 °C for 60 s. The primer sequences are listed in Table 1. The gene encoding glyceraldehyde-3-phosphate dehydrogenase (*GAPDH*) was used as the internal control, and the relative gene expression levels were calculated using the 2^−∆∆CT^ method. Each sample was analyzed in duplicate qPCR reactions.

### 2.4. Statistical Analysis

All statistical analyses were performed using R software version 4.5.2 (https://cran.r-project.org/). Because tissue samples from six cardiac regions were obtained from each animal, measurements from different regions within the same animal were treated as repeated observations. Owing to the small sample size (*n* = 3) and the repeated-measures study design, differences among cardiac regions were analyzed using the Friedman test, a non-parametric test. When a significant overall effect was detected, post hoc comparisons with the right atrium (RA) were performed using Dunnett’s test. Differences with a *p*-value of less than 0.05 were considered statistically significant. Principal component analysis (PCA) was performed to evaluate global patterns of regional gene expression across the six cardiac regions based on the expression levels of the seven target genes.

## 3. Results and Discussion

### 3.1. Histological Features of the Bovine Myocardium

**Figure 1:**
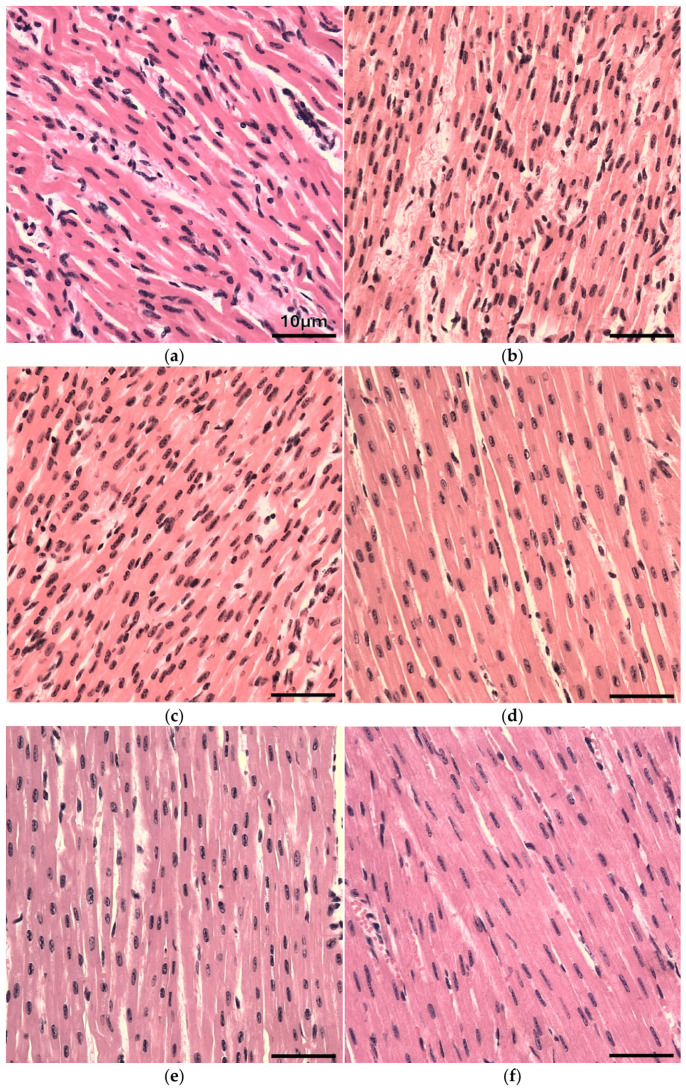
Representative images of hematoxylin and eosin (H&E) staining of (**a**) right atrium; (**b**) left atrium; (**c**) right ventricle; (**d**) left ventricle; (**e**) interventricular septum; (**f**) apex (×200; scale bar: 10 μm). No clear differences in nuclear morphology or myocardial fiber organization were observed among the regions.

Tissue sections were stained with H&E to histologically assess the region-specific structural characteristics of the bovine myocardium (Figure 1). Based on the qualitative assessment, no clear differences in nuclear morphology or myocardial fiber organization were observed among the RA, LA, RV, LV, IVS, and Apex. These findings indicate that the gross myocardial architecture is largely conserved across these regions. Therefore, functional specialization may be driven by molecular differences. 

### 3.2. Expression Levels of Marker Genes Related to Cardiac Development

**Figure 2:**
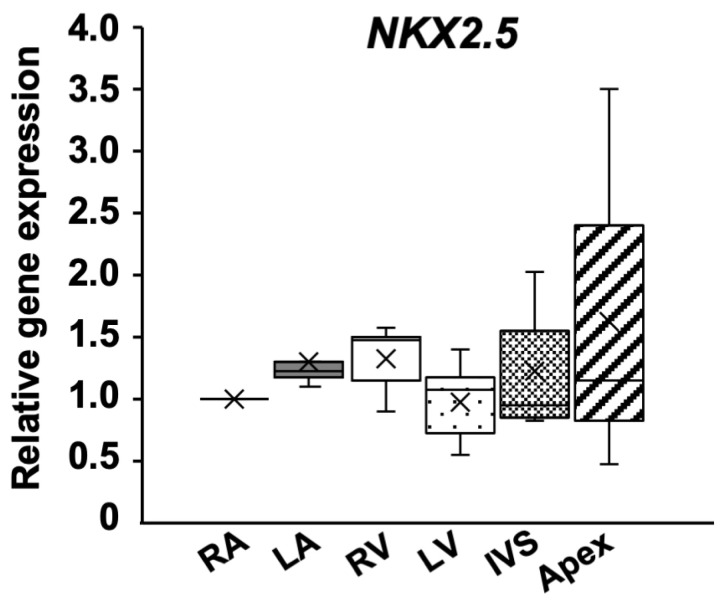
Gene expression related to cardiac development in bovine heart tissue. Bovine heart tissues were collected from the right atrium (RA), left atrium (LA), right ventricle (RV), left ventricle (LV), interventricular septum (IVS), and apex (Apex) (*n* = 3). The relative mRNA expression levels were normalized to those in the right atrium. Statistical significance was determined using the Friedman test.

To investigate regional differences in the molecular characteristics of the bovine myocardium, we compared the expression levels of the *NKX2.5* gene, which encodes a key cardiac transcription factor involved in heart development, in the RA, LA, RV, LV, IVS, and Apex. The expression levels were quantified using qPCR and normalized to the levels in the RA. No significant regional differences in *NKX2.5* expression were found. This suggests that the role of the transcription factor is conserved across the various regions of the mature heart (*p* = 0.26, Figure 2), a fact that supports its function in maintaining cardiomyocyte identity rather than driving region-specific differentiation. This indicates that the fundamental molecular identity of cardiomyocytes is broadly maintained across the various regions of the mature myocardium.

### 3.3. Expression Levels of Marker Genes Related to Myocardial Contraction

To investigate the regional differences in the molecular characteristics related to myocardial contraction and structure in bovine cardiac tissue, the expression levels of several cardiac marker genes (*MYH7*, *MYL2*, *TNNI3*, and *TNNT2*) in the six regions were analyzed. The gene expression levels were quantified using qPCR and normalized to the levels in the RA.

**Figure 3:**
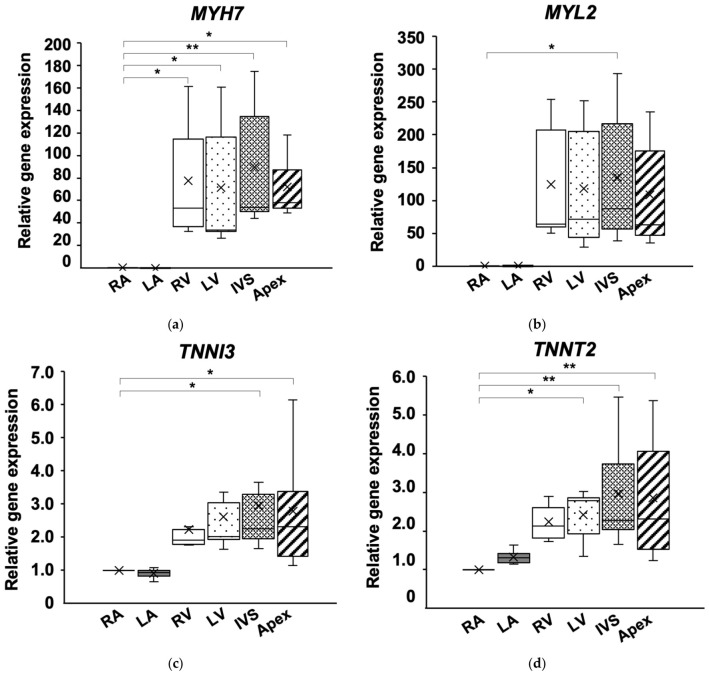
Gene expressions related to myocardial contraction in bovine heart tissue. Relative gene expression levels of (**a**) *MYH7*; (**b**) *MYL2*; (**c**) *TNNI3*; (**d**) *TNNT2*. Bovine heart tissues were collected from the right atrium (RA), left atrium (LA), right ventricle (RV), left ventricle (LV), interventricular septum (IVS) and apex (Apex) (*n* = 3). The relative mRNA expression levels were normalized to those in the RA. Statistical significance was determined using the Friedman test followed by Dunnett’s post hoc test. * *p* < 0.05, ** *p* < 0.01.

*MYH7* and *MYL2* encode myosin proteins involved in cardiomyocyte contraction. The relative expression of *MYH7* was significantly higher in the RV (*p* = 0.02), LV (*p* = 0.04), IVS (*p* = 0.01), and Apex (*p* = 0.04) than in the RA (Figure 3a). Similarly, the expression level of *MYL2* was significantly higher in the IVS than in the RA (*p* = 0.04, Figure 3b), with a general tendency toward higher expression in ventricular myocardium than in atrial myocardium. Similarly, the expression levels of *TNNI3* and *TNNT2*, which encode components of the cardiac troponin complex, also exhibited regional differences. The expression level of *TNNI3* was significantly higher in the IVS (*p* = 0.02) and Apex (*p* = 0.03) than in the RA (Figure 3c). The expression level of *TNNT2* was significantly elevated in the LV (*p* = 0.04), IVS (*p* = 0.003), and Apex (*p* = 0.005) compared with that in the RA (Figure 3d).

These findings indicate regional heterogeneity in the expression of genes associated with contractile function and cellular structure, with consistently higher expression in the ventricular myocardium. This pattern aligns with those of previously reported region-specific gene expression profiles in humans and mice [4,6], suggesting that similar molecular heterogeneity exists in the bovine myocardium. 

### 3.4. Expression of BCAA Metabolism-Related Genes

**Figure 4:**
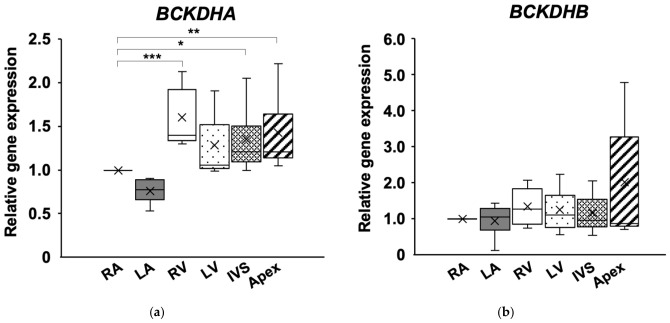
Gene expressions related to BCAA metabolism in bovine heart tissue. Relative gene expression levels of (**a**) *BCKDHA* (**b**) *BCKDHB*. Bovine heart tissues were collected from the right atrium (RA), left atrium (LA), right ventricle (RV), left ventricle (LV), interventricular septum (IVS), and apex (Apex) (*n* = 3). The relative mRNA expression levels were normalized to those in the right atrium. Statistical significance was determined using the Friedman test followed by Dunnett’s post hoc test. *: *p* < 0.05, **: *p* < 0.01, ***: *p* < 0.001.

To assess the regional differences in mitochondrial BCAA metabolism, the expression levels of genes related to this metabolic process were analyzed in the six heart regions and normalized to the levels in the RA. *BCKDHA*, which encodes the catalytic E1α subunit of the branched-chain α-keto acid dehydrogenase (BCKDH) complex, showed significant regional differences (*p* < 0.05). Compared with the RA, *BCKDHA* expression was significantly higher in the RV (*p* < 0.001), IVS (*p* = 0.04), and Apex (*p* = 0.01) (Figure 4a), indicating a general tendency toward higher expression in ventricular regions than in atrial regions. By contrast, the expression level of *BCKDHB*, which encodes the structural E1β subunit, did not differ significantly among the six regions (*p* = 0.22, Figure 4b).

Given that BCKDHA directly contributes to the activity of the enzyme [17,18], its expression is likely to be regulated in response to metabolic demand. The higher expression level observed in the ventricular myocardium may reflect greater energy demand and metabolic activity in this region. Consistent with this, regional differences in oxidative metabolism in the human heart associated with variations in workload have been reported [19]. By contrast, because BCKDHB primarily contributes to the structural stability of the BCKDH complex [20,21], the expression of its gene may be maintained at a relatively constant metabolic level. Recently, BCAA metabolism has attracted attention as a key component of cardiac metabolic regulation and a potential therapeutic target [13,18]. The present findings provide preliminary evidence that genes associated with BCAA metabolism are differentially expressed among anatomical regions of the bovine heart. Therefore, additional studies incorporating measurements of BCKDH enzyme activity, metabolite concentrations, and other markers of metabolic function will be required to determine whether the observed transcriptional differences are associated with functional differences in regional BCAA metabolism in bovine heart.

### 3.5. Principal Component Analysis of the Gene Expression Patterns

**Figure 5:**
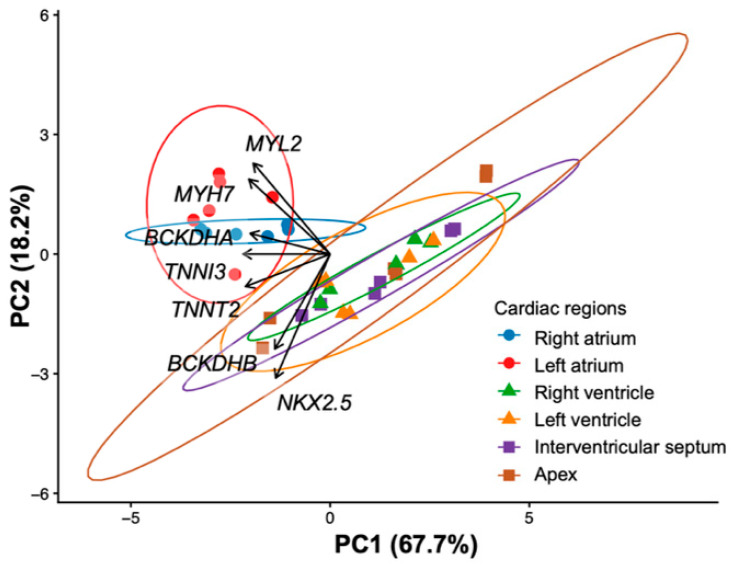
Principal component analysis (PCA) of bovine heart tissue characteristics. PCA biplot illustrating the separation of bovine heart tissue regions based on gene expression profiles.

To further evaluate the gene expression patterns in the bovine myocardium, PCA was performed using the expression data of the cardiac marker and BCAA metabolism-related genes. The proportions of variance explained by the first (PC1), second (PC2), and third principal components (PC3) were 67.7%, 18.2%, and 7.9%, respectively, with a cumulative contribution of 93.8% (Table 2). PC1 was characterized by high absolute loadings of cardiac contractile (*TNNI3*, *TNNT2*, *MYH7*, and *MYL2*) and BCAA metabolism-related (*BCKDHA* and *BCKDHB*) genes. These findings are consistent with the results of the single-gene analyses, in which these genes were more highly expressed in the ventricular myocardium. Therefore, PC1 represents an integrated axis that reflects the functional state of the myocardium, encompassing both the contractile and metabolic processes. By contrast, PC2 was characterized by negative contributions from *NKX2.5* and *BCKDHB* and positive contributions from *MYL2* and *MYH7*, suggesting that this component reflects a contrast between myocardial differentiation/metabolic processes and the contractile machinery. In the PCA score plot, the samples were primarily separated along the PC1 axis, with a clear distinction between the atrial and ventricular myocardium (Figure 5). This separation is consistent with the regional differences observed in the single-gene expression analyses and represents their multivariate integrations.

These results indicate that the atrial and ventricular myocardium in bovine hearts can be clearly distinguished on the basis of gene expression patterns, such as those of *TNNT2*, *TNNI3*, and *MYH7*. By contrast, left–right differences within the ventricles were relatively small, suggesting that the ventricular myocardium possesses comparatively consistent molecular characteristics. These findings suggested region-specific molecular differences that were not detectable by morphological assessments alone in the bovine heart. Furthermore, our findings suggest that cardiomyocyte gene expression differs among anatomical regions and cannot be fully explained by a simple atrial-versus-ventricular classification. This highlights the importance of integrating molecular biology analyses with conventional histological approaches in studies on regional heterogeneity in myocardial tissues. Future studies incorporating higher-resolution histological techniques and single-cell analyses are expected to advance our understanding of the region-specific structural and functional properties of the bovine heart. These findings provide a useful framework for future studies investigating regional myocardial specialization and functional heterogeneity in large animal hearts.

The present study provides initial evidence that gene expression differs among anatomically distinct regions of the bovine heart. Although chamber-specific gene expression patterns have been extensively characterized in humans and rodent models, comparable information remains limited in large animal species. Our findings indicate that the bovine heart exhibits clear molecular differences between atrial and ventricular regions, specifically in genes associated with contractile function and branched-chain amino acid metabolism.

These observations are important because large animal hearts differ substantially from those of rodents in terms of cardiac size, hemodynamic load, myocardial architecture, and physiological function. Therefore, characterization of regional molecular differences in the bovine heart provides valuable information for comparative cardiac biology and contributes to a broader understanding of myocardial organization across mammalian species. In particular, the elevated expression of *BCKDHA* in ventricular myocardium suggests that BCAA-related pathways may contribute to chamber-specific metabolic regulation. While fatty acid and glucose metabolism have been extensively studied in the heart, relatively little attention has been paid to regional variation in BCAA-related pathways. The present findings provide an initial basis for future investigations in this area.

Several limitations of this study should be acknowledged. First, the sample size was small (*n* = 3), reflecting the practical constraints associated with obtaining cardiac tissues from industrial animals. Although the repeated-measures study design partially mitigated inter-animal variability, the limited number of biological replicates reduced statistical power. Therefore, the present findings should be interpreted as preliminary observations requiring validation with a larger number of animals in future studies.

Second, this study employed a targeted candidate-gene approach and evaluated only a limited number of genes associated with cardiac structure, development, and BCAA metabolism. Consequently, the present data do not provide a comprehensive assessment of the bovine cardiac transcriptome, and broader transcriptomic analyses will be required to fully characterize regional molecular heterogeneity within the myocardium.

Third, the animals included in this study differed in age and genetic background. Potential effects of age, breed, and other biological variables cannot be completely excluded. Future studies using larger and more homogeneous populations will be necessary to address these factors more rigorously.

In addition, the histological evaluation performed in the present study was qualitative in nature. Quantitative morphometric analysis may provide further insights into the structural basis of the observed molecular heterogeneity. Furthermore, although differences in *BCKDHA* expression were identified, gene expression data alone cannot establish differences in BCAA enzyme activity or metabolic flux. Additional investigations incorporating enzyme activity assays, metabolomic analyses, and functional assessments will be necessary to determine the physiological significance of the observed transcriptional differences.

Taken together, the present study should be regarded as an exploratory investigation and an initial characterization of regional gene expression patterns in the bovine heart. As a short communication, the primary objective of this work was to provide foundational data and generate hypotheses for future studies rather than to comprehensively define the mechanisms underlying myocardial regionalization. Despite these limitations, the observed differences in gene expression between atrial and ventricular myocardium suggest that molecular heterogeneity exists within the bovine heart. These findings provide a foundation for future studies investigating regional myocardial biology and metabolic regulation in large animal species.

## 4. Conclusions

In this study, we suggested the existence of region-specific differences in gene expression within the bovine myocardium. The observed heterogeneity in the expression of myocardial marker and BCAA metabolism-related genes likely reflects the intrinsic differences in the functional and structural properties of atrial and ventricular cardiomyocytes. These findings provide preliminary evidence of regional molecular heterogeneity in the bovine heart. In conclusion, the present study indicates region-specific differences in cardiac marker and BCAA metabolism-related gene expression in the bovine myocardium.

## Figures and Tables

**Table 1:** Primer list.

Gene Name	Accession Number	Primer Sequence (5′-3′)	Temp (°C)	Product Length (bp)
*NKX2.5*	NM_001046443.2	F: CCTTCTATCCGCGTGCCTAT R: CAGATCTTGACCTGCGTGGA	60	283
*MYH7*	NM_174727.1	F: TCAAGGAGCTCACGTACCAG R: ACGGCTACTCCTCATTCAAGC	60	273
*MYL2*	NM_001035025.2	F: CAAGGAGATGCTGACAACGC R: TGGAGGTGGATAAATGAGGCAG	60	269
*TNNT2*	NM_174771.3	F: GTATGAGGAGCAGGAAGAAGCA R: GAAATGCGCCTCGATCAGTG	60	292
*TNNI3*	NM_001040517.1	F: CTGCAGATTGCAAAGCAGGAAC R: CAGAGTGGGCCGCTTAAACT	60	273
*BCKDHA*	NM_174506.1	F: AGGGTTTGGAGACCAAGTCG R: ATTTGAGCACCTTCTCCTGGG	60	295
*BCKDHB*	NM_174507.2	F: GCAGGTGGCTCACTTCACTT R: CATACTTGTCTCGCAAGCCG	60	197
*GAPDH*	NM_001034034.2	F: CCGTTCGACAGATAGCCGTA R: ATGACGAGCTTCCCGTTCTC	60	256

**Table 2:** Factor loadings and eigenvalues of the first three principal components for seven genes in bovine myocardium.

Gene	Factor-Loadings		
	PC1	PC2	PC3
*TNNT2*	−0.43	−0.16	0.32
*TNNI3*	−0.44	0.00	−0.12
*MYL2*	−0.39	0.46	0.05
*MYH7*	−0.41	0.38	−0.11
*NKX2.5*	−0.27	−0.62	0.50
*BCKDHA*	−0.40	0.10	0.07
*BCKDHB*	−0.28	−0.48	−0.78
Eigenvalue	4.74	1.27	0.55
Proportion (%)	67.7	18.2	7.9

## Data Availability

The data presented in this study are available from the corresponding author upon reasonable request.

## Supplementary Materials

The following supporting information can be downloaded at: https://www.mdpi.com/article/10.3390/ani16132014/s1, Table S1. Characteristics of cattle used in this study, including sex, breed, and age at sampling. Figure S1. Schematic illustration of the sampling locations in the bovine heart. Tissue samples were collected from six anatomically distinct regions: the right atrium (RA), left atrium (LA), right ventricle (RV), left ventricle (LV), interventricular septum (IVS), and apex (Apex) in bovine heart. Colored circles indicate the approximate locations of tissue collection used for histological and gene expression analyses.
