# Supplementary material for: Regional Differences in Cardiac Marker Gene Expression and Branched-Chain Amino Acid Metabolism in the Bovine Heart
Source: Animals (Basel). 2026 Jul 1;16(13):2014. doi: 10.3390/ani16132014 (PMC13359638; doi:10.3390/ani16132014)
Supplement: Supplementary file 1 [file animals-16-02014-s001.zip › animals-4376172-supplementary.pdf]

Supplemental Table

Table S1. Characteristics of cattle used in this study, including sex, breed, and age at sampling.

Animal No.	Sex	Breed	Age
1	Female	F1 (Holstein × Japanese Black)	5 months
2	Female	Holstein	2 weeks
3	Male	Holstein	3 months

Supplemental Figure

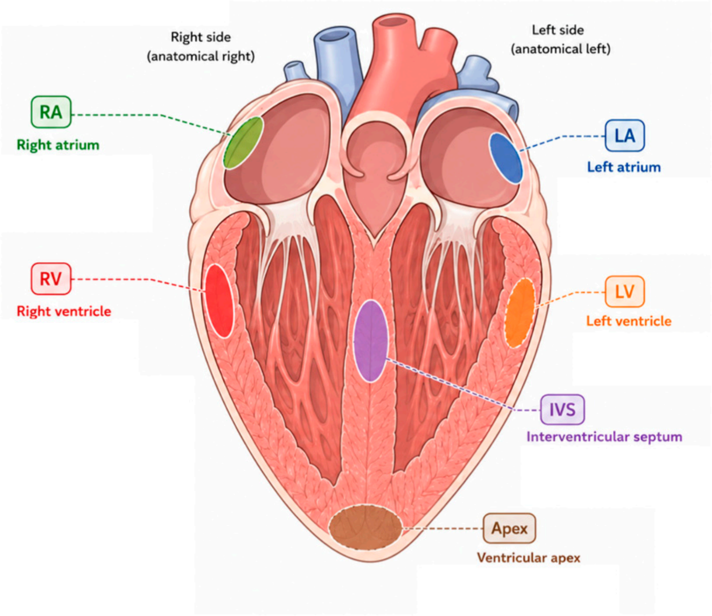

Figure S1. Schematic illustration of the sampling locations in the bovine heart.
Tissue samples were collected from six anatomically distinct regions: the right atrium (RA), left atrium (LA), right ventricle (RV), left ventricle (LV), interventricular septum (IVS), and apex (Apex) in bovine heart. Colored circles indicate the approximate locations of tissue collection used for histological and gene expression analyses.
